# Establishing Responsiveness and Minimal Clinically Important Difference of Quebec Back Pain Disability Scale (Hindi Version) in Chronic Low Back Pain Patients Undergoing Multimodal Physical Therapy

**DOI:** 10.3390/healthcare11040621

**Published:** 2023-02-20

**Authors:** Irshad Ahmad, Akhil Sharma, Sahar Zaidi, Mastour Saeed Alshahrani, Ajay Prashad Gautam, Abdullah Raizah, Ravi Shankar Reddy, Shalini Verma, Tarushi Tanwar, Mohammad Ejaz Hussain, Deepak Malhotra, Shadab Uddin, Emadeldin Mohammed Mukhtar

**Affiliations:** 1 Department of Medical Rehabilitation Sciences, College of Applied Medical Sciences, King Khalid University, Abha 61413, Saudi Arabia; 2 St. Stephens Hospital, Tis Hazari, New Delhi 110054, India; 3 Department of Physiotherapy, Jamia Hamdard, New Delhi 110062, India; 4 Department of Orthopedic Surgery, College of Medicine, King Khalid University, Abha 61413, Saudi Arabia; 5 Centre for Physiotherapy and Rehabilitation Sciences, Jamia Millia Islamia, New Delhi 110025, India; 6 Faculty of Allied Health Sciences and Physiotherapy, SGT University, Gurugram 122505, India; 7 Department of Physical Therapy, Faculty of Applied Medical Sciences, Jazan University, Jazan 82911, Saudi Arabia; 8 Department of Radiological Sciences, College of Applied Medical Sciences, King Khalid University, Abha 61413, Saudi Arabia

**Keywords:** low back pain, activity limitations, patient-reported outcome measures

## Abstract

Increasing emphasis is placed on physical functional measures to examine treatments for chronic low back pain (CLBP). The Quebec Back Pain Disability Scale (Hindi version) (QBPDS-H) has never been evaluated for responsiveness. The objectives of this study were to (1) examine the internal and external responsiveness of the Quebec Back Pain Disability Scale (Hindi version) (QBPDS-H) and (2) find out the minimal clinically important difference (MCID) and minimal detectable change (MDC) in the functional ability of patients with chronic low back pain (CLBP) undergoing multimodal physical therapy treatment. In this prospective cohort study, QBPDS-H responses were recorded at the baseline and after eight weeks from 156 CLBP patients undergoing multimodal physiotherapy treatment. To differentiate between the clinically unimproved (n = 65, age: 44.16 ± 11.8 years) and clinically improved (n = 91, age: 43.28 ± 10.7 years) scores of patients from the initial assessment to the last follow-up, the Hindi version of the Patient’s Global Impression of Change (H-PGIC) scale was utilized. Internal responsiveness was large (E.S. (pooled S.D.) (n = 91): 0.98 (95% CI = 1.14–0.85) and Standardized Response Mean (S.R.M.) (n = 91): 2.57 (95% CI = 3.05–2.17)). In addition, the correlation coefficient and receiver operative characteristics (R.O.C.) curve were used to assess the QBPDS-H external responsiveness. MCID and MDC were detected by the R.O.C. curve and standard error of measurements (S.E.M.), respectively. The H-PGIC scale showed moderate responsiveness (ρ = 0.514 and area under the curve (A.U.C.) = 0.658; 95% CI, 0.596–0.874), while the MDC achieved 13.68 points, and the MCID was found have 6 points (A.U.C. = 0.82; 95% CI: 0.74–0.88, sensitivity = 90%, specificity = 61%). This study shows that QBPDS-H has moderate levels of responsiveness in CLBP patients receiving multimodal physical therapy treatment, so it can be used to measure the changes in disability scores. MCID and MDC changes were also reported with QBPDS-H.

## 1. Introduction

Chronic low back pain (CLBP) is common among the general population and is the second leading cause of work-related disability across the globe. It is defined as chronic when the lower back pain lasts for more than 12 weeks and may be of the continuous or episodic type [1]. CLBP has an impact on both the individual’s physical functionality and quality of life [1]. Physical therapy is one of the treatment remedies used to treat CLBP [2,3]. Various tools are used to measure treatment efficacy in CLBP, and functional capacity is one such measure used in clinical settings [4]. In clinics, measuring the patients’ health-related changes due to treatment effects or differentiation of the individual differences due to different treatments is of utmost importance [5]. To assess the changes in a patient’s functional capacity, measurement tools must have good responsiveness. They should be able to find even the minute changes in functional status over time [6,7]. Furthermore, the clinical change that occurred should be sufficient enough to be referred to as an original change and accurate enough to detect minimal but necessary clinical change over time that must be meaningful for the patient [8,9]. Considering these aspects, concepts of minimal clinically important difference (MCID), along with minimal detectable change (MDC), were developed that help clinical practitioners assess and interpret the changes in the patient’s health [10]. MDC corresponds to the smallest amount of change in the patient’s score, which represent the true change, and is not due to measurement errors, whereas MCID is the minimal amount of changes in the patients’ health status outcomes, which may be considered to be meaningful by the patient or clinician.

Thorough assessment and accurate diagnosis of CLBP requires a consideration of several variables [11] pertinent to general health, pain, satisfaction, and disability [12]. Appropriate screening tools such as questionnaires, scales, range of motion baseline readings, physical examination, and strength [13] should be used, which provide evidence-based information about an individual’s risk and recovery. Questionnaires also inform us about the intervention’s efficacy and are considered to be equally reliable and valid outcome measures [14]. Various questionnaires are available to assess the disability caused by CLBP, among which the ones commonly used in clinical settings are the Oswestry Disability Index (ODI) [15], Quebec Back Pain Disability Scale (QBPDS) [16], Roland-Morris Disability Questionnaire (RMDQ) [17], and the Low Back Outcome Score [18]. These measurement questionnaires have been translated and validated in several languages. The validity and reliability of QBPDS have been established in the translated version of several languages, including the Hindi version. Even QBPDS responsiveness has been checked in a few studies in different languages such as English, Italian, French, Dutch, and Portuguese, but to our interest, was its Hindi version that has been proven to be a reliable and valid questionnaire in the Hindi-speaking population [19], but the responsiveness of it has not been established yet.

The responsiveness of an instrument is a measure of its ability to detect any clinically relevant changes over time, even if these changes are small. Responsiveness analyses a diagnostic test’s ability to quantify change over time and the main effect of the treatment. No gold standard to exemplify responsiveness exists, but according to the different methods to evaluate responsiveness, a suitable statistical measure may be used. Internal responsiveness characterizes a test’s ability to alter over a pre-specified period. In contrast, external responsiveness represents the amount of changes in an outcome over a specified duration that relates to corresponding changes in a given health status outcome. Generally, a standard instrument should be valid and reliable to be considered accurate. Sometimes, just as measurements obtained with a test may be reliable but not valid, similarly, it is possible for an analysis to yield reliable measurements, but to be non-responsive. There is inconclusive evidence about whether a test can produce unreliable measurements, yet be responsive [20,21,22].

Therefore, it is essential to check the responsiveness of a questionnaire to incorporate it in clinical and research settings to estimate and enhance the efficacy of various treatment protocols for managing CLBP. Hence, we decided to conduct this study with the aim to verify the response capacity of the QBPDS-H version and find MCID and MCD in CLBP patients undergoing multimodal physical therapy treatment.

## 2. Materials and Methods

### 2.1. Design and Participants

This questionnaire responsiveness assessment study had a cohort design, with the questionnaire responses collected initially and at the end of eight weeks. The participants were CLBP patients diagnosed by an experienced orthopedic surgeon as per standard guidelines [23], who were referred for physiotherapy treatment at Jamia Millia Islamia University, the Physiotherapy and Rehabilitation Sciences Centre, New Delhi, India. The sample size was calculated from clinical observations of the minimal clinically important differences for the response from our experience from our routine practice of patients. After performing a calculation to obtain the proportion difference, (n = Z1 − α/2)^2^ pq/d^2^) 158 was the sample, considering 20% dropouts and at a 5% level of significance.

Eligibility criteria: age between 18–65 years old; LBP must be >12 weeks with or without a history of radiation to the leg causing functional limitations (Roland Morris Disability Questionnaire 3 score) [15,17]; can walk ≥ 100 m without interruption. Exclusion criteria: not willing to complete the questionnaire, any history of musculoskeletal trauma, any pathology (inflammatory, infectious, or malignant ones) of the spine, cardiovascular or metabolic disease, pregnancy, or any severe co-morbidity such as feet gangrene/muscular dystrophies that would affect the participant’s ambulation. The study procedure was explained to the patients before their participation, and detailed explanations about the questionnaire contents meaning were given, and outcomes were obtained during their initial and final visits to our clinic, and written informed consent was obtained as per the Declaration of Helsinki after receiving approval from the Institutional Ethics Committee (Proposal No.: 31/10/178/JMI/IEC/2018).

### 2.2. Study Protocol

Before the initial testing, each patient was given a screening form for a primary health evaluation that also contained demographic details such as age, gender, education level, occupation, and body mass index. The patients were asked to fill out QBPDS-H, the Hindi version of the Roland-Morris Disability Questionnaire (H-RMDQ), and the Visual Analogue Scale (VAS) at the first visit. All participants received an individually tailored similar multimodal physical therapy rehabilitation program of electrotherapy (Interferential current), thermotherapy (moist heat), and muscular motor training exercises targeting trunk core muscles stabilization and strength, along with stretching, for one hour. However, the treatment given and the gap between the assessments helped only as a construct for achieving a change [24,25]. It was neither a part of this study’s interests, nor was it considered. The follow-up evaluation was performed after eight weeks of the rehabilitation program, including QBPDS-H, H-RMDQ, VAS, and the H-PGIC scale. Based on their rating on the Hindi version of the Patient’s Global Impression of Change scale (H-PGIC), the patients were dichotomized into two subgroups, and ratings of 1–4 were categorized as “clinically unimproved”, while ratings of 5–7 were categorized as “clinically improved” [26]. The changes in QBPDS-H scores (∆QBPDS-H) were calculated for each subgroup as the differences in scores between the baseline and at the end of the eighth week. A positive score indicated functional ability improvement. The percentage change in scores was calculated by dividing the change by the original value and multiplying it by a hundred ((∆QBPDS-H/QBPDS-H baseline) × 100). Overall changes experienced in their CLBP status were also acquired using the H-PGIC scale, which was achieved by observing how significant differences occurred in their pain-related disability in activities of daily living from the baseline and after eight weeks.

### 2.3. Questionnaires

The QBPDS is a self-reported measure that evaluates LBP patients’ functional status about “today” on a twenty-item scale of primarily daily activities that will be affected by LBP, which corresponds to six categories of responses, i.e., 1. bed/rest items, 2. sitting/standing items, 3. ambulation items, 4. movement items, 5. bending/stooping items, and 6. the handling of large/heavy objects items. All items are scored from 0 (No difficulty) to 5 (not able to do), which, when summed up, provides a total score. On this scale, the score range is from 0–100, with low total scores corresponding from no disability and higher scores corresponding to a serious disability [19]. The PGIC scale is a seven-point transition scale scored from 1 to 7, ranging from no change to a considerable improvement, which was developed to find patients feeling an overall improvement of their back pain. Both scales’ validation and cross-cultural adaptations have been established and are available in Hindi and other languages [19,26,27]. RMDQ (24-point scale) is a valid and reliable tool to assess mild-to-moderate disability and is a self-administered measure, where higher disability levels are reflected by higher numbers, and it is sensitive to patients with LBP (acute, sub-acute, and chronic types), and the patient has to choose the response that is applicable to them on that particular day [15,17], whereas VAS is the most widely used measure to assess the pain, and this scale tries to measure the amount of pain that a patient feels ranges across a continuum from none to an extreme amount of pain. VAS is a reliable and valid tool to assess CLBP.

### 2.4. Data Analysis

Data were analyzed using SPSS software (version 24.0; I.B.M., Chicago, IL, USA). Descriptive statistical methods were used for explaining the demographic characteristics. Each data subset was initially tested for the normal distribution of scores, and non-parametric tests were employed for non-normal data. We used the Mann–Whitney test to compare the baseline scores between two groups (clinically unimproved and clinically improved ones) and the Wilcoxon test to compare QBPDS-H pre-post scores. Spearman’s rank-order correlation assessed the association between ∆QBPDS-H and H-PGIC to evaluate the responsiveness of QBPDS-H. COSMIN recommendations were followed for the evaluation of the proposed measures [28].

Responsiveness was assessed in two ways, i.e., the distribution way, which assess the ability to detect changes, and the anchor way includes the H-PGIC Scale as an anchor to find the clinical meaningful changes in the outcome scores. The external responsiveness was assessed in terms of sensitivity and specificity of QBPDS-H, which was determined using receiver operating characteristics (R.O.C.), and the area under the curve (A.U.C.) was used to predict the probability of correctly discriminating between the two groups. The Minimum Clinically Important Difference (MCID) was valued for raw and % scores by finding the closest point to the left upper corner of the R.O.C. curve [29].

Another indicator of internal responsiveness is the effect size, which reflects the amount of change in any measure. It was calculated as a standardized effect size (E.S.) (from pooled S.D.), as well as a standardized response mean (S.R.M.), using the MedCalc program, version 12.21 (MedCalc Software, Mariakerke, Belgium). S.R.M., also known as the Responsiveness Treatment (R.T.) coefficient/efficacy index, uses values of ≤0.2 as minimal, 0.5 as moderate, and ≥0.8 as large responsiveness [30]. The intra-class correlation coefficient (ICC_2,1_) was estimated for the QBPDS-H pre- and post-scores in the “clinically unimproved” subgroup to compute the standard error of measurement (S.E.M.) and the minimum detectable change (MDC) for repeated measures. Additionally, MDC was utilized to determine the scale width and to assess its floor and ceiling effects [30,31], and a *p*-value of ≤ 0.05 level was considered to be statistically significant.

## 3. Results

**Figure 1:**
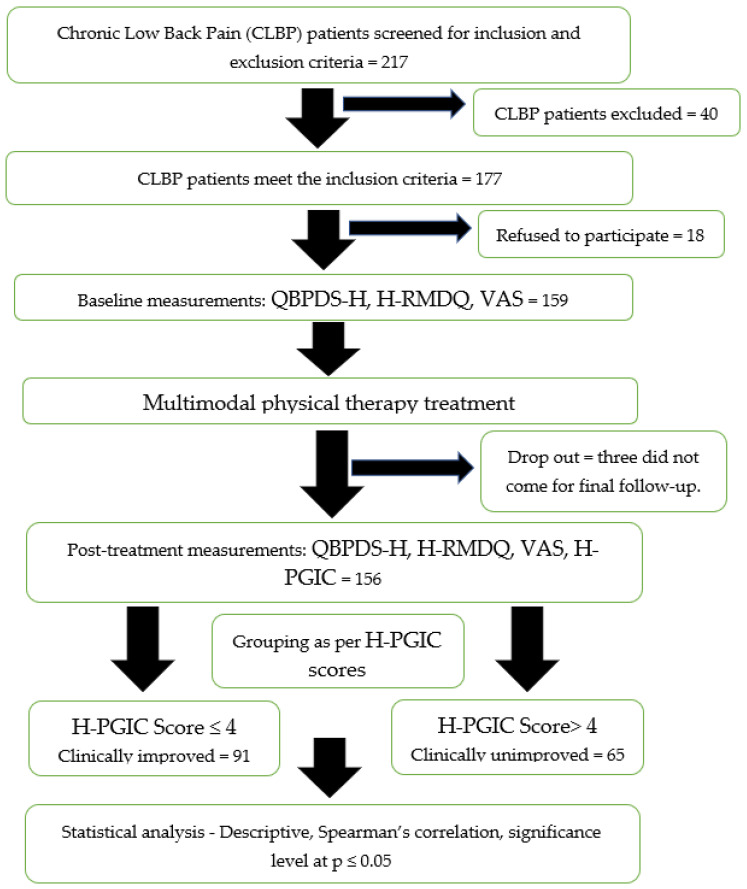
Participants’ progression throughout the study.

The participants’ progression in the study is explained in Figure 1. Patients with CLBP were screened for inclusion and exclusion criteria and among 217 screened patients, 177 patients met the required inclusion criteria, and 40 were excluded. Among 40 excluded patients, 23 had history of radiating pain, 12 had previous history of spine trauma/surgery, 2 were unable to walk 100 m, and 3 were not willing to complete the questionnaires. Out of those patients who met inclusion criteria, only 159 agreed to participate, and in the end, 156 completed the final responses and were used for the data analysis. The patients were divided based on the final H-PGIC scores into two groups: “clinically unimproved (≤4 scores)” (n = 65; 41.7%) and “clinically improved (>4 scores)” ones (n = 91; 58.3%).

The above mentioned table show descriptive statistics of the demographic characteristics of the clinically unimproved and clinically improved people that were found to be comparable at the study entry (Table 1). Additionally, the QBPDS-H baseline scores were comparable in both of the groups. In this study, there were 53.4% male and 46.6% female participants, and almost all the participants had an education level above higher secondary school level, and 89% were employed.

The above-mentioned table describes the QBPDS-H scores at the baseline and after multimodal physical therapy rehabilitation. The Wilcoxon test results (Table 2) demonstrated that the QBPDS-H scores for the “clinically unimproved” group did not show a significant change from the baseline to the end of follow-up (*p* = 0.169). However, there was statistically significant change from the baseline to the end of follow-up (*p* < 0.001) in the QBPDS-H scores for the “clinically improved” group.

**Figure 2:**
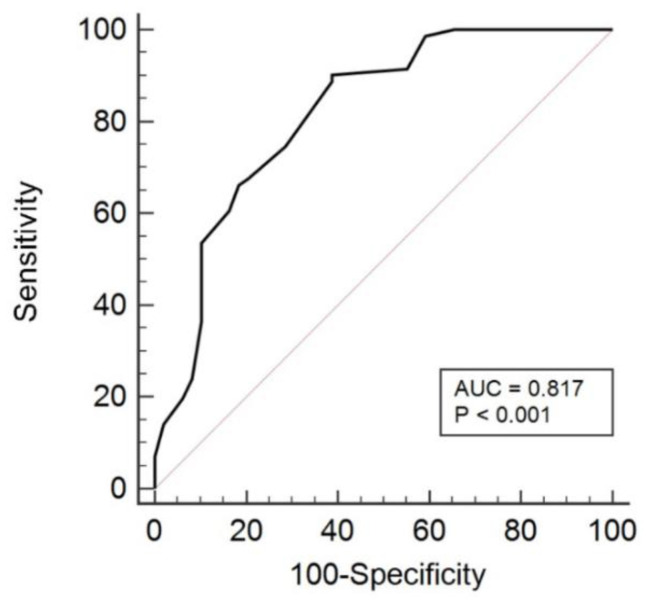
R.O.C. analysis of QBPDS-H—A.U.C. and optimal cut-off value.

The above-mentioned figure shows the R.O.C. analysis of the absolute changes in the scores, and also, the optimal cut-off values of QBPDS-H (Figure 2). For external responsiveness, the Spearman rank coefficient, i.e., ρ value, came to 0.592 (*p* < 0.001), indicating a moderate, positive correlation between the scores of ∆QBPDS-H and H-PGIC, and it was statistically significant. The optimal cut-off value of the QBPDS-H was six points (A.U.C. = 0.82 (95% CI: 0.74–0.88), with a specificity of 61.2% and sensitivity of 90.1%) (Figure 2). An alternative R.O.C. analysis depending upon the QBPDS-H % change in the relative score from the initial assessment showed an optimal cut-off value of 21% (A.U.C. = 0.75 (95% CI: 0.66–0.83), sensitivity was 94.4%, and specificity was 53.1%).

The responsiveness of QBPDS-H for clinically improved patients evaluated through E.S. was found to be 0.98 (95% CI: 1.14–0.85), and the S.R.M. was estimated to be 2.57 (95% CI: 3.05–2.17), which is substantially high. Although greater values show a greater specificity and sensitivity, no criteria concerning the cut-off values to define a suitable level of responsiveness exists.

The above-mentioned table describes the test–retest reliability indices and distribution of the parameters of responsiveness of the QBPDS: H version (Table 3). Absolute reliability, which is indicative of the precision of the scores on repeated measurements, was demonstrated by S.E.M. and MDC95%. The intraclass correlation coefficient estimated for the clinically stable group was found to be ICC_2,1_ = 0.92, which yielded an S.E.M. = 4.94 and MDC 95% = 13.68 (Table 3). The scores of repeated measurements must be consistent in a study. I.C.C. reflects an estimation of the relative reliability of the measurements’ consistency and a test’s ability to distinguish between patients and their position relative to the other group. I.C.C. = 0.770 and I.C.C. = 0.92 were observed for the clinically improved and clinically stable groups, respectively. This I.C.C. was used to calculate the S.E.M. and MDC.

MDC, which is context specific by nature, uses different external criteria for each perspective differently. An MDC of 95% of almost 14 points was determined for QBPDS-H. So, it was estimated that the width of the QBPDS-H scale is between 14 and 86. The baseline scores were found to be below 14 for two patients, whereas scores above 86 were not reported, suggesting that no floor or ceiling effects were observed in this study sample.

## 4. Discussion

For the measurement of the clinical changes in the patients’ health status, any tools used for assessing the outcomes the responsiveness, MCID, and MDC play an important role in the patients’ care and clinical research. Researchers use these parameters to calculate the sample size, statistical power setting, as well as finding the cost of given treatments and the progression of the disease. Additionally, these parameters help in clinical practice for finding the effectiveness of given treatments and formulating the clinical guidelines for clinical decision making [28]. QBPDS-H is a valid and reliable tool for assessing the functional status in our CLBP patient’s population, and this study primarily aimed to identify the internal, as well as external, responsiveness, and also, the MCID of the QBPDS-H in CLBP patients undertaking multimodal physiotherapy treatment. Our study is the only research which evaluated the clinical responsiveness and MCID of QBPDS-H in Indian CLBP patients receiving multimodal physical therapy. This study’s findings postulated that the QBPDS-H is a responsive measure to evaluate the minimal change in functional disability, and also, to establish the MDC, along with the MCID in CLBP patients. These attributes make the QBPDS-H a clinically relevant tool that may be particularly well suited for the functional status assessment of CLBP patients, thus ruling out disability. There are various methods such as distribution and/or anchor procedures, with which we can detect the responsiveness and MCID, where the former one tells us about the magnitude of change, but it lacks the clinical importance of observed changes, while latter method is better at calculating the MCID.

There are a few studies that have estimated the measurement properties of QBPDS for chronic LBP. QBPDS has been interpreted in various languages, and its reliability and validity have also been established [29,32,33]. The responsiveness of QBPDS in some translated versions has been checked [34,35,36]. The distribution of the E.S. and S.R.M. analysis found that QBPDS-H has a moderate level of responsiveness in our patients, and similar results were reported by few previous researchers [29,36]. For the anchor-type analysis, there was a moderately positive, statistically significant correlation between ∆QBPDS-H and H-PGIC, and another finding of this study was a significant change in the pre- and post-QBPDS-H scores for clinically improved patients. In contrast, there was no significant improvement in the clinically stable patients. There were similar results findings reported by other researchers for the Italian and Portuguese versions of QBPDS [29,36]. This shows that as the QBPDS-H scores decrease with an increase in H-PGIC, this reflects better health of the CLBP patients.

In our study the responsiveness of QBPDS for clinically improved patients evaluated through E.S. was found to moderate to high, and the S.R.M. was estimated to be substantially large. Although greater values show a greater specificity and sensitivity, no criteria concerning the cut-off values to define a suitable level of responsiveness exists. Any variability within the population and differences in the baseline values may influence the responsiveness of a measure [37]. The ODI responsiveness range is 9–14, and the RMDQ range is 2.5–5 [15,38]. These data are also similar to those of other available studies, which showed values for the ODI ranges of 4–23 in cases of subacute/chronic LBP [6], 4–15 in cases of acute/chronic LBP [39], and 12.8 in post-operative cases [40]. In contrast, our study shows that the responsiveness (minimal detectable change) of the Hindi version of QBPDS is 13.68, which suggests that the difference of 13.68 points in the pre- and post-scores of QBPDS is a significant change. These MDC values indicate that a change in the score that is smaller than its values are extraneous for the patient; however, a greater value may indicate an improvement in the patient’s conditions [20].

The R.O.C. analysis reports suggest that QBPDS-H has a good capacity to grade CLBP patients that have clinically improved or not clinically improved. By comparing the baseline to the follow-up scores, an absolute ideal cut-off score of 6 points was obtained using the R.O.C. analysis, and this was the MCID for our patients population, which is higher than that of Demoulin et al. (5 points) [24], equal to that of Monticone et al. (6 points) [38] and less than those of Roer et al. (8.5 points) [41] and Viera et al. (6.5 points) [29]. For the analysis, the A.U.C. was 0.82 (*p* < 0.001), which was comparatively smaller than those of Fritz et al. (0.87) [42], Demoulin et al. (0.85) [23], and Monticone et al. (0.83) [38] and higher than those of Viera et al. (0.74) [20] and Davidson et al. (0.74) [43]. These MDC values represent that if a variation in the score is smaller than its original value, it must be viewed as irrelevant to the patient. However, variations in the score of more than the range suggest that the treatment resulted in an improvement of the patient’s condition [29].

The result of the present study showed that the S.E.M. was 4.94 and the MDC was 13.68. The MDC, which is context specific by nature, uses different external criteria for each perspective differently. In a previous report, it was established that the MDC of QBPDS was from 17.5 to 32.9 and 8.5 to 24.6 for sub-acute and chronic patients with LBP, respectively. The MDC for patients with sub-acute LBP was significantly larger than that for patients with chronic LBP, regardless of the method used [39]. From the above analysis even the results were relatively consistent for the MCID and MDC; if the MCID scores are smaller than the MDC scores, it will be a problem, as the MDC has to be considered as a starting step for establishing the MDIC by keeping it as a benchmark of any measurements errors. In clinical practice, changes that are greater than the attained MDIC, i.e., 6 points, but smaller than the MDC, i.e., 13.8 points, suggests an improvement. In our study, by considering the percentage changes in the QBPDS-H scores from the baseline, the R.O.C. analysis showed an optimal cut-off value of 21%, which is in line with the suggested cut-off values of 20–30% by expert panels [44] and other cut-off values of 18-24% reported in studies that are similar to our study [24,29,36].

Considering the overall results, we suggest that percent change in QBPDS-H scores from the baseline values must be very important while assessing the effectiveness of multimodal physical therapy in CLBP patients [24,29,36]. Additionally, we also agree with the previous expert’s opinion [44] that MCID should be calculated in different LBP patient’s populations in various clinical scenarios.

### 4.1. Clinical Significance

Questionnaires, nowadays, have the advantage of being used as an assessment, prognostic, and diagnostic tool, which is less time consuming and gives the desired result. Therefore, calculating the responsiveness of the QBPDS-H aimed to provide the clinicians and researchers with a subjective tool that would help them to assess the functional disability and improvement of the functional status of CLBP patients. Additionally, we have established the MCID and MDC for our population, which can be used in the Indian CLBP patient population.

### 4.2. Limitations and Future Recommendations

Questionnaires are a subjective assessment method, therefore, some clinicians do not consider them to be valid tools. The sample was from a single center, hence, the results are not generalized for the whole country’s population. Stable patients were recruited for the study; patients with underlying specific pathology were not included. Since we have taken the non-specific LBP population to maintain the homogeneity of the research, one should assess the responsiveness of this scale in specific conditions such as S.I. dysfunction, Lumbar Canal stenosis, etc. This will help to establish the Hindi version of QBPDS as a gold standard for assessing functional disability in any LBP condition. Additionally, some additional factors may affect the responses for effectiveness of the treatment such as the psychological status and quality of life of the patient, so these must also be considered in future research.

## 5. Conclusions

Our study of the QBPDS-H version found that is has good interpretability and is responsive to changes in functional disability following treatment in our CLBP patient’s population undergoing multimodal physical therapy. It is as good as an objective measurement of functional disability and can detect MDC effectively. From a clinical perspective, QBPDS has the advantage of practicality, and it is a time-saving technique to assess the functional disability and it has minimal chances of incurring errors because it is easy to understand and is filled by the patient themselves. Thus, QBPDS-H may be used in research and clinical settings as a diagnostic and prognostic tool for functional disability in multimodal physical therapy rehabilitation.

## Figures and Tables

**Table 1:** Baseline comparison of the demographic and clinical characteristics between the two groups.

Variable	Clinically Unimproved Group Mean (S.D.) (n = 65)	Clinically Improved Group Mean (S.D.) (n = 91)	t	*p*-Value
Age (years)	44.16 (11.8)	43.28 (10.7)	0.424	0.672
Weight (kg)	70.16 (12.6)	72.01 (8.4)	0.421	0.675
Height (cm)	162.29 (10.3)	163.10 (7.3)	0.901	0.371
BMI (kg/m^2^)	26.45 (2.6)	27.07 (2.4)	1.299	0.197
QBPDS-H pre	36.06 (17.6)	38.46 (13.8)	0.703	0.482

BMI = body Mass Index, QBPDS-H pre = Baseline Score of Quebec Back Pain Disability Scale: Hindi Version, t = Z-score from Mann–Whitney test.

**Table 2:** Comparison of change in QBPDS-H scores in the two groups (Wilcoxon test).

Variable	Group	Baseline	Post-Intervention	*p*-Value
QBPDS-H	Clinically unimproved (n = 65)	36.06 (17.6)	33.45 (18.9)	0.169
	Clinically improved (n = 91)	38.46 (13.8)	26.01 (11.5)	<0.001 *

QBPDS-H = Quebec Back Pain Disability Scale: Hindi version, * Significant Difference.

**Table 3:** Reliability analysis of QBPDS-H scores in the clinically unimproved group.

QBPDS-H ^1^	Mean (S.D.) (n = 65)	ICC_2,1_^2^	SEM ^3^	MDC 95% ^4^
Pre	36.06 (17.6)	0.92	4.94	13.68
Post	33.45 (18.9)			

^1^ QBPDS-H = Quebec Back Pain Disability Scale: Hindi version, ^2^ ICC_2,1_ = Intraclass Correlation Coefficient, ^3^ SEM = Standard Error of Measurement, ^4^ MDC = Minimum Detectable Change.

## Data Availability

All data generated or analyzed during this study are included in in the given manuscript.
